# Association of intradialysis blood sodium level, blood pressure variability, and hydration status with hemodialysis-related headache: a prospective cohort study

**DOI:** 10.1186/s10194-023-01701-2

**Published:** 2023-12-11

**Authors:** Yuqin Xiong, Nujia You, Ruoxi Liao, Ling Wu, Yao Liu, Ziying Ling, Yang Yu

**Affiliations:** 1 Kidney Research Institute, West China Hospital of Sichuan University, Chengdu, China; 2 Department of Nephrology, West China School of Public Health and West China Fourth Hospital, Sichuan University, Chengdu, China; 3 China School of Nursing, Sichuan University, Chengdu, China; 4 Department of Nephrology, Kidney Research Institute, West China Hospital of Sichuan University, Chengdu, China; 5 Department of Nephrology, West China Hospital, Sichuan University, No. 37, Guoxue Alley, Wuhou District, Chengdu, Sichuan Province China

**Keywords:** Hemodialysis, Headache, Blood pressure, Blood sodium, Blood gas analysis, Body composition

## Abstract

**Objective:**

To identify primary factors contributing to hemodialysis-related headache (HRH) in maintenance hemodialysis (MHD) patients.

**Methods:**

Adult outpatients receiving MHD were prospectively enrolled from a hemodialysis (HD) center of a tertiary hospital in China. Twelve dialysis sessions were successively monitored for each patient. HRH is defined as having at least three headache episodes that begin during HD and resolve within 72 h of HD session completion. Blood gas analysis during headache episodes and body composition analysis after dialysis were conducted. Hour-to-hour vital sign variability during dialysis was assessed using the metric of average real variability (ARV). Multivariable logistic regression analysis was conducted to explore the factors triggering HRH.

**Results:**

A total of 95 Chinese MHD patients were enrolled, with 92 patients (60.9% were males) included in the final analysis. The mean age of the 92 patients was 59.3 ± 17.5 years, and the median dialysis vintage was 27.1 (12–46.2) months. Among them, 12 patients (13%) complained of 42 headache attacks, and eight (8.7%) were diagnosed with HRH. For eight patients with HRH, headache occurred 100.3 ± 69.5 min after the start of dialysis, with a mean VAS score of 4.3 ± 1 points. The quality of headaches was dull (six patients), pulsating (one patient), or stabbing pain (one patient); all the headaches were bilateral, with one having concomitant vomiting. The intradialysis headache duration and the whole headache duration were 98.8 ± 68.1 and 120 (65–217.5) minutes, respectively. Younger age (OR = 0.844, 95% CI 0.719–0.991, *p* = 0.039), decreased blood sodium level (OR = 0.309 in the range of 133–142 mmol/L, 95% CI 0.111–0.856,* p* = 0.024), increased ARV of intradialysis systolic blood pressure (OR = 3.067, 95% CI 1.006–9.348, *p* = 0.049) and ratio of overhydration to dry weight (OR = 1.990, 95% CI 1.033–3.832, *p* = 0.040) were found to be independent risk factors for HRH.

**Conclusions:**

This study suggested a significant attribution of blood sodium, hydration status and blood pressure variability to HRH.

**Supplementary Information:**

The online version contains supplementary material available at 10.1186/s10194-023-01701-2.

## Background

Headache is a commonly reported neurological symptom in patients undergoing maintenance hemodialysis (MHD) [1–7]. According to the criteria of the International Headache Society third edition (ICHD III), hemodialysis-related headache (HRH) refers to a headache that starts during dialysis, resolves within 72 h of dialysis session completion, and ceases after successful transplantation [8]. Previous studies, using the ICHD II criteria (2004), reported that HRH occurs in 6.6–70% of patients [1–6], with a recent study showing a 41.6% frequency according to the ICHD III beta version criteria [7].

The pathophysiology of HRH remains multifactorial and controversial. Past studies identified possible contributors, such as differences in blood urea, arterial hypertension and hypotension, sodium washout, an elevated predialysis sodium level, or a decreased magnesium level [1–6]. However, existing studies have limitations [1–7]. First, blood biochemical measures were taken randomly, making it difficult to reflect the biochemical conditions during headache episodes. Second, most studies have focused on the effect of pre- and/or postdialysis blood pressure (BP) on HRH, neglecting the role of intradialysis fluctuations in vital signs. Third, the association between volume status (overhydration, normal hydration, poor hydration) and dialysis-related adverse effects is well known, but studies on HRH have rarely quantified the volume status of patients. Last, most studies explored triggering factors for HRH by comparing clinical features between the HRH group and the control group rather than using regression analyses to identify independent influencing factors. Therefore, the primary factors contributing to HRH need a more thorough evaluation.

The aim of the present study was to determine the dominant factors for HRH in MHD patients with blood biochemistry measurements during headache episodes, assessments of intradialysis vital sign variability, and volume assessment based on body composition analysis.

## Patients and methods

### Study population

The single-center prospective cohort study was conducted at the Hemodialysis Center of the West China Fourth Hospital of Sichuan University, a public tertiary hospital affiliated with Sichuan University in China. Study participants were recruited between October 17, 2022 and February 24, 2023. The inclusion criteria were (1) outpatients aged 18 years or older and (2) received regular hemodialysis (HD) for at least 3 months. The exclusion criteria were as follows: (1) peritoneal dialysis; (2) a previous history of primary or secondary headaches (e.g., adverse drug reactions) with attacks occurring more than 50% of the time between HD sessions, which was assessed through clinical interview and electronic medical records; (3) dementia, cognitive impairment**,** or consciousness disorder preventing cooperation with investigators; (4) active severe infection, e.g., intracranial infection, severe pneumonia; (5) admission to hospital for any reason; (6) having schedules of kidney transplant operations or transferring dialysis center within 2 months after the enrollment day such that the study had to be suspended; and (7) refusal to consent to the study.

### Clinical data collection

Clinical information was gathered through electronic medical records, including demographic details, etiologies of end-stage renal disease, coexisting diseases, dialysis vintage and HD prescription. Serum biochemical indexes were obtained based on the blood samples measured in a central laboratory from a routine clinical examination in the HD center, which was gathered within three months, close to the enrollment day. The urea reduction ratio (URR) was defined as the percentage drop in blood urea nitrogen levels from before to after a dialysis session, which was calculated using the simple formulation 1-(post-urea nitrogen/pre-urea nitrogen) × 100 (%) [9].

### Hemodialysis procedure monitoring

Patients in this study underwent 2–3 acetate HD treatments per week. Two different types of anticoagulation, including low molecular weight heparin anticoagulation and citrate anticoagulation, were employed for a 4-h HD treatment according to the patient’s clinical condition. Blood flow was 200‐350 mL/min, and dialysate flow was 500‐700 mL/min (dialysate: sodium 138‐140 mmol/L, potassium 2 mmol/L, calcium 1.25–1.5 mmol/L, magnesium 0.5 mmol/L, chlorine 108.5 mmol/L, HCO3- 31 mmol/L). The ultrafiltration volume was determined by interdialysis weight gain.

HD treatments (12 sessions per patient) over a period of 4–6 weeks were consistently recorded, including data on ultrafiltration volume, dialysate flow, blood flow, and vital signs such as pulse, BP, and blood oxygen saturation (SpO_2_) of non-fistula limbs throughout the dialysis session (at 0 h, 1 h, 2 h, 3 h, and 4 h). There were no interventions by the investigators.

### Hemodialysis-related headache definition and measurements

For each headache attack during HD treatment, the patient underwent physical and neurological examinations by a physician and responded to a semi-structured questionnaire detailing headache characteristics, including onset time, location, duration, quality and concomitant symptoms [1, 10] (Supplementary files 1 and 2). The intensity of pain was assessed using the visual analog scale (VAS), graded from 0 to 10 points. Interventions and their effects on headache were also documented. According to the ICHD-III criteria (ICHD-3 code 10.2), HRH is defined as having at least three headache episodes that start during HD and resolve within 72 h of HD session completion [8].

For patients diagnosed with HRH, pre-filtration blood samples (0.2 ml) were collected at the time of the headache attack and then subjected to blood gas analysis (BGA) using the same handheld blood gas analyser (i-STAT1, Abbott Point of Care Inc., USA). Body composition analysis (BCA) using the same body composition monitor (BCM; Fresenius Medical Care, Germany), following the machine manual [11], was performed 0.5 to 1 h after completing the dialysis session that had triggered the headache attack. BCA data regarding overhydration volume (in litres) and dry weight (in kilograms) were acquired, and the absolute value of the ratio of overhydration to dry weight (|OH/DW|) was calculated to indicate the degree of hydration imbalance normalized by dry weight.

For patients without HRH, BGA and BCA were performed during and after a routine HD treatment, respectively. The measuring time of BGA in the control group was consistent with the most common onset time of headaches in the HRH group.

### Vital sign variability assessment and statistical analysis

Variability assessment of hourly vital signs during HD treatment employed the average real variability (ARV) metric, representing the mean of the absolute differences between successive measurements. ARV offers a notable advantage, as it considers not only the amplitude of fluctuations in the study target but also the time sequence of these fluctuations [12, 13]. For each patient, the formula for ARV is as follows: ARV = $\frac{{\sum }_{\mathbf{K}=1}^{\mathbf{N}-1}|{(\overline{\mathbf{P}\mathbf{u}\mathbf{l}\mathbf{s}\mathbf{e}}/\overline{\mathbf{B}\mathbf{P}}/\overline{\mathbf{S}\mathbf{p}\mathbf{O}2})}_{\mathbf{K}+1}-{(\overline{\mathbf{P}\mathbf{u}\mathbf{l}\mathbf{s}\mathbf{e}}/\overline{\mathbf{B}\mathbf{P}}/\overline{\mathbf{S}\mathbf{p}\mathbf{O}2})}_{\mathbf{K}}|}{\mathbf{N}-1}$, where the $\overline{\mathrm{pulse}}$, $\overline{\mathrm{BP}}$, or $\overline{\mathrm{SpO}2}$ refers to the mean values of pulse, BP, or SpO2 at 0, 1, 2, 3, 4 h on 12 dialysis sessions, N is the number of pulse, BP or SpO2 measurements, and K is the order of a measurement [12, 13].

Study patients were divided into two groups according to the diagnosis of HRH. Cases with missing values for certain variables were excluded from analyses including the variable. Continuous variables are presented as the mean with standard deviation or median with interquartile range for nonnormally distributed variables. Categorical variables are presented as percentages. The two-sample *t* test or Wilcoxon rank sum test was used to compare continuous variables between the HRH and non-HRH groups, and the chi-square test was applied to compare proportions. Univariable and multivariable logistic regression analyses were conducted with the dependent variable defined as a patient having HRH. Variables at *P* < 0.05 in the univariate analysis and those considered clinically relevant to HRH based on prior studies (e.g., BP, homeostasis, blood sodium) [1–8] or that were aimed to be clarified in this study (e.g., variability metrics of vital signs, |OH/DW|) were entered into the multivariate regression model, with odds ratio (OR) and 95% confidence interval (CI) reported. The Hosmer‒Lemeshow test and classification table were employed to explore the goodness-of-fit and reliability of the relatively optimal models, with *p* > 0.05 regarded as an acceptable model. The level of significance was set to a two-sided *p* value of < 0.05. Data were analysed using STATA 11.0 MP (StataCorp, www.stata.com) and SPSS version 23.0 (IBM, NY, USA).

## Results

**Fig. 1:**
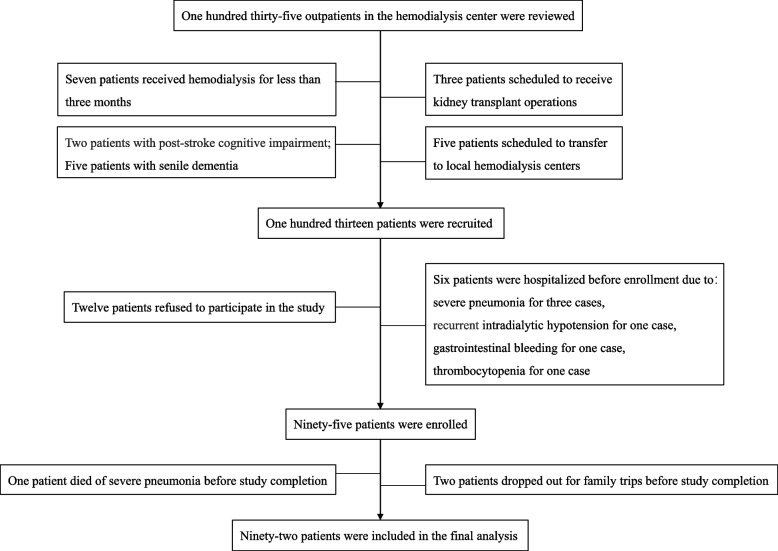
Flow diagram of the process of enrollment and exclusion of study patients

Ninety-five Chinese MHD outpatients were enrolled from the HD center that administered 135 MHD patients. The investigation period was between November 28, 2022 and April 10, 2023. Among the study patients, three patients dropped out due to one death from severe pneumonia, and two travelled before study completion. A total of 92 patients were included in the final analysis, with two patients lacking values in post-urea nitrogen (Fig. 1). The mean age of the 92 patients was 59.3 ± 17.5 years, among whom 60.9% were males. The main etiologies of end-stage renal disease were chronic immune glomerulonephritis or nephropathy (35.9%), diabetic glomerulopathy (20.7%), and hypertensive nephropathy (20.7%). The median dialysis vintage was 27.1 (12–46.2) months, and the permanent vascular access for HD was arteriovenous fistula (64.1%), arteriovenous graft (1.1%) and tunneled cuffed catheter (34.8%). The prevalence rates of cerebrovascular disease, coronary heart disease and diabetes were 14.1%, 6.5% and 37.0%, respectively.

Twelve of 92 patients (13%) reported a total of 42 headache attacks in the total 1104 HD sessions, among whom eight (8.7%) were diagnosed with HRH (Table 1). For the eight patients with HRH, headache occurred 100.3 ± 69.5 min after the start of dialysis. The localization of pain was bitemporal in four patients, vertex in three patients, occipital in three and bifrontal in two patients; all eight patients had bilateral headaches. The headache duration during HD and the whole headache duration were 98.8 ± 68.1 and 120 (65–217.5) minutes, respectively. The mean VAS score was 4.3 ± 1 points. The quality of headaches was dull (six patients), pulsating (one patient), or stabbing pain (one patient); one of the patients had headaches with concomitant vomiting; none of the headaches was aggravated by routine physical activity. For treatment, one patient received an ultrafiltration suspension and BP control, and two patients received intravenous 50% glucose; however, the pain was not remitted effectively.

**Table 1:** Patients with headache attack during hemodialysis and diagnosis of hemodialysis-related headache

Cases	HD with headache among 12 HD sessions no. (%)	Mean time to develop headache after the start of HD	Mean headache duration during HD	Mean whole headache duration	Meets definition of HRH^a^
Case 1	3 (25.0)	200 min	40 min	70 min	Yes
Case 2	2 (16.7)	60 min	180 min	180 min	No
Case 3	4 (33.3)	180 min	60 min	120 min	Yes
Case 4	4 (33.3)	120 min	120 min	120 min	Yes
Case 5	2 (16.7)	180 min	60 min	90 min	No
Case 6	3 (25.0)	120 min	120 min	120 min	Yes
Case 7	8 (66.7)	92 min	120 min	315 min	Yes
Case 8	4 (33.3)	0 min	240 min	690 min	Yes
Case 9	4 (33.3)	60 min	60 min	60 min	Yes
Case 10	5 (41.7)	30 min	30 min	30 min	Yes
Case 11	2 (16.7)	60 min	30 min	30 min	No
Case 12	1 (8.3)	0 min	240 min	240 min	No

*Abbreviations*: *HD* hemodialysis, *HRH* hemodialysis-related headache

^a^According to the ICHD-III criteria (ICHD-3 code 10.2), HRH is defined as having at least three headache episodes that start during HD and resolve within 72 h of HD session completion

As shown in Table 2, patients with HRH had a higher systolic BP (SBP, 150.1 ± 19.6 *vs.* 136.7 ± 14.2 mmHg, *p* = 0.016) and diastolic BP (DBP, 86.5 ± 15.4 *vs.* 73.5 ± 11.1 mmHg, *p* = 0.05) at HD-0 h than those without HRH. Compared to the non-HRH group, the HRH group showed a significantly lesser SPO2-ARV (0.2 ± 0.1 *vs.* 0.4 ± 0.2, *p* = 0.044) and an insignificantly increased SBP-ARV (6.1 ± 2.8 *vs.* 5.5 ± 2.3, *p* = 0.495). For BGA, the HRH group had a slightly lower blood sodium level (136.8 ± 2.2 *vs.* 137.7 ± 1.8 mmol/L, *p* = 0.156) than the non-HRH group. For hydration assessment, the HRH group showed a nearly double |OH/DW| than that in the non-HRH group (243.5 (74.5–538.2) *vs.* 132 (73.8–204.5) %, *p* = 0.329). In the multivariable regression analysis (Table 3), age (OR = 0.844 per 1-year increase, 95% CI 0.719–0.991, *p* = 0.039), SBP-ARV (OR = 3.067 per 1-point increase, 95% CI 1.006–9.348, *p* = 0.049), blood sodium level (OR = 0.309 per 1 mmol/L increase in the range of 133–142 mmol/L, 95% CI 0.111–0.856, *p* = 0.024), and |OH/DW| (OR = 1.990 per 1% increase, 95% CI 1.033–3.832, *p* = 0.040) were found to be independent risk factors for HRH.

**Table 2:** Characteristics in groups according to hemodialysis-related headache

Characteristic mean ± SD, no. %), or median (IQR)	Total	Non hemodialysis- related headache	Hemodialysis-related headache	*P *value
No. of patients	92	84	8	-
Age (year)	59.3 ± 17.5	60.4 ± 17.5	47.9 ± 12.8	0.052
Male	56 (60.9)	51 (60.7)	5 (62.5)	1.000
History of cerebro-vascular diseases	13 (14.1)	13 (15.5)	0 (0.0)	0.503
Dialysis vintage (month)	27.1 (12.0–46.2)	27.1 (12.3–46.2)	27.8 (5.3–44.6)	0.618
URR (%)	65.1 ± 10.9	64.7 ± 11.2	68.5 ± 8.3	0.351
Predialysis blood biochemistry examination				
Hemoglobin (g/L)	111.1 ± 20.6	111.1 ± 21.0	111.6 ± 16.7	0.944
Albumin (g/L)	42.1 ± 4.3	41.9 ± 4.4	43.7 ± 2.6	0.270
Sodium (mmol/L)	138.9 ± 3.1	138.9 ± 3.1	138.5 ± 2.9	0.702
Potassium (mmol/L)	4.9 ± 0.6	4.9 ± 0.6	4.9 ± 0.9	0.994
Calcium (mmol/L)	2.2 ± 0.2	2.2 ± 0.2	2.3 ± 0.3	0.937
Magnesium (mmol/L)	1.0 ± 0.2	1.0 ± 0.2	1.1 ± 0.1	0.461
Mean vital signs at hemodialysis-0 h for 12 dialysis sessions				
Pulse (beats/min)	81.2 ± 9.4	81.4 ± 9.5	79.5 ± 9.3	0.599
SBP (mmHg)	137.9 ± 15.1	136.7 ± 14.2	150.1 ± 19.6	0.016
DBP (mmHg)	74.6 ± 12.0	73.5 ± 11.1	86.5 ± 15.4	0.050
SpO2 (%)	97.2 ± 0.7	97.2 ± 0.7	97.3 ± 0.6	0.782
Mean hourly variability of vital signs during dialysis				
Pulse-ARV	2.9 ± 1.3	2.9 ± 1.3	3.1 ± 1.0	0.647
SBP-ARV	5.6 ± 2.4	5.5 ± 2.3	6.1 ± 2.8	0.495
DBP-ARV	3.7 ± 1.6	3.7 ± 1.5	3.9 ± 2.3	0.673
SpO2-ARV	0.3 ± 0.2	0.4 ± 0.2	0.2 ± 0.1	0.044
Blood gas analysis				
pH (%)	742.2 ± 4.9	742.2 ± 4.9	742.5 ± 5.1	0.840
pCO2 (mmHg)	39.0 ± 4.8	38.9 ± 4.7	39.6 ± 5.8	0.693
Base excess (mmol/L)	0.8 ± 1.8	0.8 ± 1.8	1.3 ± 2.1	0.498
HCO3- (mmol/L)	25.4 ± 2.1	25.4 ± 2.1	26.0 ± 1.8	0.382
Sodium (mmol/L)	137.7 ± 1.9	137.7 ± 1.8	136.8 ± 2.2	0.156
Potassium (mmol/L)	3.6 ± 0.5	3.6 ± 0.5	3.3 ± 0.7	0.138
Ion calcium (mmol/L)	1.2 ± 0.1	1.2 ± 0.1	1.2 ± 0.1	0.773
Glucose (mmol/L)	5.9 ± 2.1	5.9 ± 2.1	5.5 ± 2.3	0.553
**|**OH/DW**|**^a^ (%)	132.9 (73.8–211.8)	132.0 (73.8–204.5)	243.5 (74.5–538.2)	0.329

*Abbreviations*: *SD* standard deviation, *IQR* interquartile range, *URR* urea reduction ratio, *SBP* systolic blood pressure, *DBP* diastolic blood pressure, *SpO2* blood oxygen saturation, *ARV* average real variability, *pCO2* partial pressure of carbon dioxide, *OH* overhydration, *DW* dry weight

^**a**^Absolute value of the ratio of overhydration volume (in litres) to dry weight (in kilograms), which reflects the degree of hydration imbalance such as volume overload and excess ultrafiltration

**Table 3:** Risk factors for hemodialysis-related headache in logistic regression analysis

Variable	OR (univariate)	95%CI (univariate)	*P* value (univariate)	OR (multivariate)	95%CI (multivariate)	*P* value (multivariate)
Age (/1 year)	0.958	0.918–1.000	0.049	0.844	0.719–0.991	0.039
URR (/1%)	1.034	0.964–1.110	0.347	1.139	0.969–1.339	0.114
Dialysis vintage (/1 month)	0.992	0.959–1.027	0.644	0.980	0.927–1.036	0.485
Pulse-ARV (/1%)	1.141	0.671–1.941	0.627	1.279	0.471–3.473	0.629
SBP-ARV (/1 point)	1.114	0.824–1.505	0.483	3.067	1.006–9.348	0.049
DBP-ARV (/1 point)	1.105	0.707–1.728	0.660	0.479	0.178–1.294	0.147
SpO2%-ARV (/1 point)	< .001	< .001–0.660	0.038	< .001	< .001–12.901	0.140
pH (/1%)	1.017	0.873–1.184	0.829	4.463	0.648–30.718	0.129
pCO2 (/1 mmHg)	1.027	0.885–1.192	0.726	3.649	0.712–18.709	0.121
Base excess (/1 mmol/L)	1.141	0.761–1.710	0.523	0.045	0.002–1.252	0.068
HCO3- (/1 mmol/L)	1.116	0.855–1.455	0.420	2.245	0.805–6.260	0.122
Sodium (/1 mmol/L)	0.762	0.516–1.127	0.174	0.309	0.111–0.856	0.024
**|**OH/DW**|**^a^ (/1%)	1.373	0.989–1.905	0.058	1.990	1.033–3.832	0.040
Constant				< .001		0.163

*Abbreviations*: *OR* odd ratio, *CI* confidence interval, *URR* urea reduction ratio, *ARV* average real variability, *SBP* systolic blood pressure, *DBP* diastolic blood pressure, *pCO2* partial pressure of carbon dioxide, *OH* overhydration, *DW* dry weight. The dependent variable was defined as a patient having HRH. For multivariate logistic regression analysis: Hosmer–Lemeshow *χ*2 = 4.255, *p* = 0.833; percentage correct 96.7%

^**a**^Absolute value of the ratio of overhydration volume (in litres) to dry weight (in kilograms), which reflects the degree of hydration imbalance

## Discussion

The current study revealed a relatively low incidence of HRH compared to prior investigations, which might be attributed to variations in the observation period and advancements in dialysis techniques and prescriptions in recent years [1–7]. Despite the significant association between HRH and a lower quality of life [14], there exists no consensus on its treatment due to its diverse and complex causes [1–6]. This study applied a regression model with a high accuracy rate (96.7%) after incorporating relevant clinical variables related to dialysis complications, such as BP, hydration status, blood electrolytes, and URR. Consequently, younger age, lower pre-ultrafiltration blood sodium level, increased |OH/DW| and intradialysis variability of SBP were identified as significantly associated with an elevated risk of HRH.

Limited information is available regarding the impact of blood sodium alteration on HRH, although two studies indicated a higher predialysis serum sodium level (*p* = 0.003) [3] or a potentially greater difference between pre- and postdialysis sodium levels (*p* > 0.05) [4] in patients with HRH. This study addressed this issue by monitoring real-time sodium levels during headache attacks, revealing an approximately 70% reduction in the risk of HRH for every 1 mmol/L increase within a range of 133–142 mmol/L in blood sodium. Moreover, this study suggested a considerable influence of hydration imbalance on HRH (OR = 1.99, *p* = 0.04), underlining the significance of volume assessment and management in patients on MHD. In addition, these findings explained why empirical treatments, such as adjusting the dialysate sodium concentration for BP control and intravenous administration of glucose, had little effect on HRH. Specifically, lowering the dialysate sodium concentration may trigger or worsen headaches during HD. Although the blood glucose level in the HRH group was slightly lower than that in the non-HRH group (one HRH patient had an extremely low blood glucose of 2 mmol/L), it was not the primary contributor to HRH, as suggested in the regression analysis.

BP fluctuations over time are denoted as BP variability (BPV). MHD patients often experience increased BPV during HD due to the rapid removal of metabolic substances, vasoactive mediators, and body fluids, leading to dramatic alterations in hemodynamics and the internal environment [12, 13, 15]. Previous studies have demonstrated that intradialysis BPV significantly raises cardiovascular and all-cause mortality in HD patients [16, 17], and this study contributes to the literature by establishing a close positive relationship between intradialysis BPV and the occurrence of HRH. Influencing factors for intradialysis BPV include younger age, dialysis using a central venous catheter, relatively low predialysis BP, vascular calcification, antihypertensive medications, and dialysis time (morning, afternoon, or evening) [17, 18]. Among these, calcium-channel blockers were associated with lower BPV compared to non-calcium channel blocker therapy or no antihypertensive drugs. The progression of vascular calcification in CKD was found to be closely linked to hyperphosphatemia, calcium load, hypomagnesemia, iron deficiency, lipoprotein(a) abnormalities, protein malnutrition, and vitamin K deficiency [19]. Therefore, practical control of intradialysis BPV by addressing reversible influencing factors, such as adjusting the antihypertensive medication regimen and dialysis time, promoting fistula use, and delaying vascular calcification, may effectively prevent HRH.

The present study has some limitations. First, the sample size was small, and the relatively low incidence of HRH resulted in an uneven sample size between the HRH and non-HRH groups, which inevitably weakened the reliability of the differences between the two groups. Second, although pre-filtration BGA was well-accepted by patients due to advantages such as being free of venipuncture, requiring a small specimen, and providing rapid feedback of results, it did not measure blood magnesium, which is closely correlated with HRH [3]. Third, the study population, consisting of Chinese patients from an HD center in a tertiary hospital, may not reflect a nationwide or worldwide cohort, and the generalizability of the results needs to be cautioned.

## Conclusions

The prospective cohort study revealed an 8.7% incidence of HRH in MHD patients over a 12-HD session observational period based on the latest ICHD III criteria. Factors associated with an increased risk of HRH include younger age, a relatively low blood sodium level, heightened variability of intradialysis SBP, and imbalanced hydration. Further large-scale, high-quality studies are necessary to comprehensively understand the contributing factors and develop effective treatment strategies for HRH.

### Supplementary Information

**Additional file 1.** 

## Data Availability

All data generated or analysed during this study are included in this published article.

## Abbreviations

MHD: Maintenance hemodialysis
ICHD: International classification of headache disorders
HRH: Hemodialysis-related headache
BP: Blood pressure
HD: Hemodialysis
URR: Urea reduction ratio
SpO_2_: Blood oxygen saturation
VAS: Visual analog scale
BGA: Blood gas analysis
BCA: Body composition analysis
|OH/DW|: Absolute value of the ratio of overhydration to dry weight
ARV: Average real variability
OR: Odds ratio
CI: Confidence interval
SBP: Systolic blood pressure
DBP: Diastolic blood pressure
BPV: Blood pressure variability
