# Supplementary material for: Association of intradialysis blood sodium level, blood pressure variability, and hydration status with hemodialysis-related headache: a prospective cohort study
Source: J Headache Pain. 2023 Dec 11;24(1):166. doi: 10.1186/s10194-023-01701-2 (PMC10712180; doi:10.1186/s10194-023-01701-2)
Supplement: Supplementary file 1 — Additional file 1. [file 10194_2023_1701_MOESM1_ESM.zip › _____.doc]

**透析相关头痛问卷调查表**

姓名：	性别：	年龄：

填写过程中如有疑问，请与工作人员沟通，感谢您的配合。

1、头痛发作日期_________________

2、头痛当日透析班次_______

A.上午	B.下午	C.晚上	D.其他

3、头痛在透析后________分钟开始发作

4、头痛发作侧_______

A.左侧	B.右侧	C.双侧	D.其他

5、头痛发作部位（可多选）_______

A.前额部（参见图a）	B.头顶（参见图b）	C.颞部（参见图c）

D.枕部（参见图d）	E.弥散或位置不固定	F.其他

a

b

c

d

6、头痛性质（可多选）_______

A.搏动性疼痛	B.压榨痛、紧箍感	C.刺痛、刀割样疼痛	D. 胀痛、闷痛

E.其他

7、头痛是否随日常活动（如走路，爬楼梯）加重_______

A.是	B.否	C.其他

8、头痛发作形式_______

A.持续性头痛	B.阵发性头痛	C. 间断性头痛	D.其他

9、头痛伴随症（可多选）_______

A.无	B.恐惧	C.恐音	D.畏光	E.眩晕和/或眼花	F. 视物模糊

G.恶心	H.呕吐	I.其他

10、针对头痛或其并发症的治疗/处理措施（透析中及透析后）

A.无任何治疗/处理措施

B.医疗措施

C.其他措施

11、头痛经治疗/处理后的缓解程度________

A.完全缓解	B.部分缓解且缓解程度≥50%	C.部分缓解且缓解程度＜50%

D.无缓解	E.无缓解且加重	F.其他

12、透析过程中头痛持续时间_______分钟

13、本次头痛持续总时间（包括下机后的头痛时间）_______分钟

14、VAS评分（请联系工作人员协助使用VAS量尺评估）_______分

附图. VAS法疼痛评分尺
