# Supplementary material for: Association of intradialysis blood sodium level, blood pressure variability, and hydration status with hemodialysis-related headache: a prospective cohort study
Source: J Headache Pain. 2023 Dec 11;24(1):166. doi: 10.1186/s10194-023-01701-2 (PMC10712180; doi:10.1186/s10194-023-01701-2)
Supplement: Supplementary file 1 — Additional file 1. [file 10194_2023_1701_MOESM1_ESM.zip › Questionnaire.doc]

**Hemodialysis Headache Questionnaire**

Name：	Gender：	Age：

(If you have any questions in answering the following questions, please contact the staff. Thank you for your cooperation.)

1. Date of headache onset _________________

2. Time of hemodialysis session _______

A. morning	B. afternoon	C. night	D. other

3. The headache occurs ________ minutes after the start of dialysis

4. Side of the headache _______

A. left side	B. right side	C. both sides	D. other

5. Location of the headache (multiple choices allowed) _______

A. frontal (figure a)	B. vertex (figure b)	C. temporal (figure c)

D. occipital (figure d)	E. diffuse or unfixed site	F. other

a

b

c

d

6. Headache quality (multiple choices allowed) _______

A. pulsating	B. pressing or tightening	C. stabbing or cutting	D. dull

E. other

7. Headache is aggravated by routine physical activity (e.g., walking, climbing stair) ______

A. yes	B. no	C. other

8. Type of headache attack _______

A. persistent	B. paroxysmal	C. intermittent	D. other

9. Concomitant symptoms of headache (multiple choices allowed) _______

A. none	B. phobia	C. phonophobia	D. photophobia	E. dizziness	F. blurred vision	G. nausea	H. vomiting	I. other

10. Treatments/measures for headache or the complications during and after hemodialysis session (multiple choices allowed)

A. none

B. medical measures (please listed)

C. others (please listed)

11. Alleviation of headache after treatments/measures ________

A. complete remission	B. partial remission (≥50%)	C. partial remission (＜50%)	D. no remission	E. no remission and aggravation	F. other

12. Headache duration during dialysis _______minutes

13. Total headache duration during and after dialysis _______ minutes

14. VAS score for the intensity of headache (please contact medical staff for assistance with VAS scale assessment) _______ points

Appended picture. VAS pain scale
